# Conceptual grounding of language in action and perception: a neurocomputational model of the emergence of category specificity and semantic hubs

**DOI:** 10.1111/ejn.13145

**Published:** 2016-02-09

**Authors:** Max Garagnani, Friedemann Pulvermüller

**Affiliations:** ^1^ Brain Language LaboratoryDepartment of Philosophy and HumanitiesFreie Universität BerlinHabelschwerdter Allee 4514195BerlinGermany; ^2^ Centre for Robotics and Neural Systems (CRNS)University of PlymouthPlymouthDevonUK

**Keywords:** cell assembly, cortical connectivity, functional differentiation, Hebbian learning, word meaning

## Abstract

Current neurobiological accounts of language and cognition offer diverging views on the questions of ‘where’ and ‘how’ semantic information is stored and processed in the human brain. Neuroimaging data showing consistent activation of different multi‐modal areas during word and sentence comprehension suggest that all meanings are processed indistinctively, by a set of general semantic centres or ‘hubs’. However, words belonging to specific semantic categories selectively activate modality‐preferential areas; for example, action‐related words spark activity in dorsal motor cortex, whereas object‐related ones activate ventral visual areas. The evidence for category‐specific and category‐general semantic areas begs for a unifying explanation, able to integrate the emergence of both. Here, a neurobiological model offering such an explanation is described. Using a neural architecture replicating anatomical and neurophysiological features of frontal, occipital and temporal cortices, basic aspects of word learning and semantic grounding in action and perception were simulated. As the network underwent training, distributed lexico‐semantic circuits spontaneously emerged. These circuits exhibited different cortical distributions that reached into dorsal‐motor or ventral‐visual areas, reflecting the correlated category‐specific sensorimotor patterns that co‐occurred during action‐ or object‐related semantic grounding, respectively. Crucially, substantial numbers of neurons of both types of distributed circuits emerged in areas interfacing between modality‐preferential regions, i.e. in multimodal connection hubs, which therefore became loci of general semantic binding. By relating neuroanatomical structure and cellular‐level learning mechanisms with system‐level cognitive function, this model offers a neurobiological account of category‐general and category‐specific semantic areas based on the different cortical distributions of the underlying semantic circuits.

## Introduction

Current semantic theories offer diverging perspectives on how word meaning is acquired, represented and processed in the human brain. One tradition views the cognitive basis of meaning as a symbolic, ‘amodal’ system containing abstract representations defined in terms of semantic features or correlations between words, bearing no explicit relationship with the concrete objects and actions the symbols are used to speak about (Collins & Loftus, 1975; Potter, 1979; Ellis & Young, 1988). A putative brain basis for such a system of symbolic‐conceptual representations has been attributed to ‘semantic hubs’, higher‐association multimodal areas located in frontal, temporal and parietal cortices that have been found active during, or even to be necessary for, semantic processing (Price, 2000; Bookheimer, 2002; Devlin *et al*., 2003; Vigneau *et al*., 2006; Patterson *et al*., 2007; Binder & Desai, 2011; Pulvermüller, 2013).

A second tradition builds on the insight that semantic knowledge requires grounding in the real world (Searle, 1980; Harnad, 1990). Symbols are used to speak about specific objects, actions and other entities; access to such semantic knowledge likely involves processing sensorimotor information in modality‐preferential areas of the cortex: for example, understanding an object‐related word such as ‘cat’ should reactivate visual areas, whereas an action word like ‘grasp’ motor ones. Support for modality‐specific semantic processes comes from neuropsychological and neuroimaging studies showing semantic‐category specificity of cortical activations and category‐specific deficits after lesions in modality‐preferential areas (Shallice, 1988; Martin, 2007). For example, word and sentence comprehension induce category‐specific activations in modality‐preferential motor and sensory (visual, auditory, olfactory and gustatory) areas (Barsalou, 2008; Binder & Desai, 2011; Kiefer & Pulvermüller, 2012; Pulvermüller, 2013; Kemmerer, 2015).

Here, we attempt to explain and integrate the above experimental data and theories by means of a single neurobiological model. Our hypothesis is that the semantic‐category‐specific and ‐general functional behaviours observed in distinct cortical areas are a direct consequence of well‐established neuroscience facts and principles, and should therefore spontaneously emerge in specific parts of the cortex as a result of sensorimotor correlations and associative learning. To address this hypothesis, we implemented a neurocomputational model of relevant primary, secondary and higher‐association areas in the frontal, temporal and occipital lobes of the human brain and simulated elementary processes of language acquisition in it, focusing specifically on the semantic grounding of object‐ and action‐related words.

A range of previous connectionist models successfully addressed aspects of language learning and processing, although most did not attempt to replicate the neuroanatomy of the cortical areas concerned with the corresponding brain processes (Elman *et al*., 1996; Plunkett, 1997; Dell *et al*., 1999; Plaut & Gonnerman, 2000; Christiansen & Chater, 2001). While some recent works did take connectivity structure into account (Husain *et al*., 2004; Guenther *et al*., 2006; Ueno *et al*., 2011), they either did not incorporate learning mechanisms, or made use of ones (e.g. back‐propagation) whose neurobiological plausibility is questionable (Mazzoni *et al*., 1991; Braitenberg & Schüz, 1998; O'Reilly, 1998). By contrast, here we implemented only learning mechanisms that mimic well‐documented neurophysiological phenomena of Hebbian synaptic plasticity (Artola & Singer, 1993), so as to show how associative learning and neuroanatomical structure interact to bring about the two different functional behaviours in semantic processing described above (category‐specific and ‐general) in distinct cortical areas. Similar approaches have previously been used to provide neurobiological accounts for the spontaneous emergence and cortical topography of resting state activity, perceptual and action decisions, and working memory (Deco *et al*., 2013a,b; Garagnani & Pulvermüller, 2013; Pulvermüller & Garagnani, 2014).

## Materials and methods

We take a semantic grounding perspective (Barsalou, 1999; Pulvermüller, 1999) and postulate that learning the meaning of at least a basic set of words and symbols of any language involves the formation of referential‐semantic links between their ‘form’ – the articulatory‐ and acoustic‐phonological patterns in the case of single spoken words – and the types of object or action these symbols are typically used to speak about (Barsalou, 2008; Pulvermüller & Fadiga, 2010; Glenberg & Gallese, 2012; Pulvermüller, 2013). Accordingly, we use a neurocomputational model of relevant peri‐ and extra‐sylvian cortical areas (see below) to simulate the spontaneous emergence of such associative links.

### General structure and features of the model

**Figure 1:**
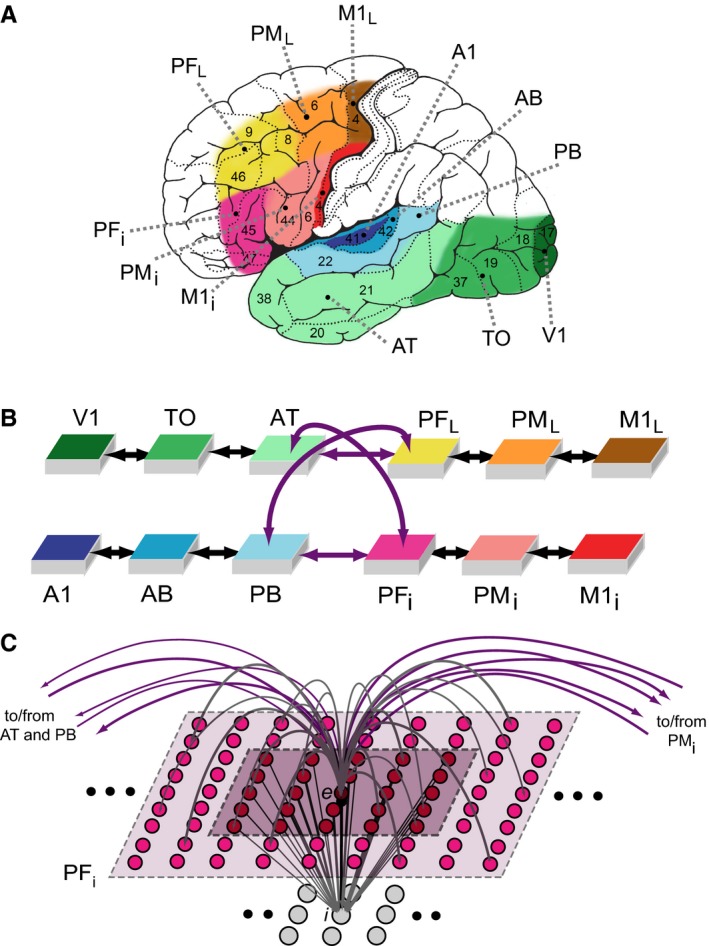
Model of lexical and semantic mechanisms. The 12 cortical areas modelled (A), their global connectivity architecture (B), and aspects of the micro‐structure of their connectivity (C) are illustrated. (A) Six perisylvian and six extrasylvian areas are shown, each including a dorsal (frontal) and a ventral (temporal) part. Perisylvian areas include three areas in inferior frontal gyrus (red colours), including inferior‐prefrontal (PF_*i*_), premotor (PM_*i*_) and primary motor cortex (M1_*i*_), and three areas in the superior temporal lobe (in blue), including auditory parabelt (PB), auditory belt (AB) and primary auditory cortex (A1). These areas can store correlations between neuronal activations carrying articulatory‐phonological and corresponding acoustic‐phonological information, for example when phonemes, syllables and spoken word forms are being articulated (activity in M1_*i*_) and acoustic features of these spoken words are simultaneously perceived (stimulation of primary auditory cortex, A1). Extrasylvian areas include three areas in lateral/superior frontal cortex (yellow to brown), including dorsolateral prefrontal (PF_*L*_), premotor (PM_*L*_) and primary motor cortex (M1_*L*_), and three areas forming the occipito‐temporal (‘what’) visual stream of object processing (green), including anterior‐temporal (AT), temporo‐occipital (TO) and early visual areas (V1). Together with the perisylvian ones, these extrasylvian areas can store correlations between neuronal activations carrying semantic information, for example when words are used (activity in all perisylvian areas) to speak about objects present in the environment (activity in V1, TO, AT) or about actions the individual engages in (activity in M1_*L*_, PM_*L*_, PF_*L*_). Numbers indicate Brodmann areas. (B) Schematic illustration of all 12 modelled areas and the known between‐area connections implemented. The colours indicate correspondence between cortical and model areas. See text for a detailed description of the neuroanatomical evidence supporting the implemented connectivity structure. (C) Schematics of micro‐connectivity of one of the 7500 single excitatory neural elements modelled (labelled ‘*e*’). Within‐area excitatory links (in grey) to and from ‘cell’ *e* are random and sparse, and limited to a local (19 × 19) neighbourhood (light‐pink shaded area). Lateral inhibition between *e* and neighbouring excitatory elements is realised as follows: the underlying cell ‘*i’* inhibits *e* in proportion to the total excitatory input it receives from the 5 × 5 neighbourhood (dark‐purple shaded area); by means of analogous connections (not depicted), *e* inhibits all of its neighbours. Each pair (*e*,* i*) of model cells is taken to represent an entire cluster or column (grey matter under approximately 0.25 mm^2^ of cortical surface) of pyramidal cells and the inhibitory interneurons therein. See Appendix A for a complete specification of the model.

The neural model consists of 12 identical interconnected ‘areas’ of graded‐response cells, implementing random and sparse between‐ and within‐area connections (Fig. 1B and C; Appendix A). Each model area consists of two layers (or ‘banks’) of excitatory and inhibitory cells, and simulates a specific cortical area (Fig. 1A).

1. As information in the articulatory motor cortex relates to the production of a word form, and information in the auditory cortex to the acoustic perception of such a form, both of these perisylvian areas (labelled M1_*i*_ and A1, respectively) were included. Moreover, as information about the objects about which we speak when using words such as ‘sun’ comes in through the primary visual cortex, and because a self‐performed action related to the meaning of action‐related words such as ‘grasp’ or ‘run’ is controlled by the lateral and superior motor cortex, the model also included primary visual and dorsolateral motor cortices (areas V1 and M1_*L*_).
2. In addition to primary cortices, ‘higher’ secondary and multimodal regions known to have strong neuroanatomical links with the above four primary sensorimotor cortices were modelled (see ‘Network structure and connectivity of the simulated brain areas’ below). These were secondary inferotemporo‐occipital visual, auditory belt, and inferior and lateral premotor cortex (TO, AB, PM_*i*_, PM_*L*_) and, respectively, adjacent multimodal anterior‐temporal, superior‐temporal (auditory parabelt) and inferior and dorsolateral prefrontal cortices (AT, PB, PF_*i*_, PF_*L*_).

The architecture builds upon and extends an existing six‐area model of the left perisylvian language cortex that was developed to simulate the emergence of memory traces for (meaningless) spoken words in the cortex and explain neurophysiological responses to linguistic stimuli (Garagnani *et al*., 2007, 2008; Garagnani & Pulvermüller, 2011). As in previous versions of the architecture, all functional and structural features implemented closely reflect well‐documented properties of the human cortex, including the following:

1. known structure of the neuroanatomical links between the modelled sensorimotor and multimodal brain systems;
2. sparse, patchy and topographic between‐ and within‐area connections, with probability of a synaptic link existing between two cells falling off with their distance (Kaas, 1997; Braitenberg & Schüz, 1998);
3. local lateral (mutual) inhibition (Fig. 1C) and area‐specific global regulation mechanisms (Braitenberg, 1978b; Yuille & Geiger, 2003);
4. Hebbian learning mechanisms, simulating synaptic plasticity phenomena of long‐term potentiation and depression (Artola & Singer, 1993);
5. neurophysiological dynamics of single cells including temporal summation of inputs, sigmoid transformation of membrane potentials into neuronal outputs, and adaptation (Matthews, 2001);
6. presence of uniform white noise (simulating spontaneous, baseline neuronal firing) in all parts of the network at all times (Rolls & Deco, 2010).

A detailed description of the connectivity structure [point (1) above] is provided in ‘Network structure and connectivity of the simulated brain areas’ below. The neural‐level features [points (2–6)] are identical to those implemented in previous versions of the model (Garagnani *et al*., 2008, 2009b; Garagnani & Pulvermüller, 2011, 2013; Pulvermüller & Garagnani, 2014). For completeness, they are summarized again in Appendix A.

Note that we strived to model only mechanisms that have a physiological correlate, and implemented a connectivity structure that closely reflects known neuroanatomical pathways between the modelled cortical areas. A direct comparison of the effectiveness and biological accuracy of the learning rule used here with that of other, known brain‐inspired synaptic plasticity rules is provided in Garagnani *et al*. (2009b). Although the implementation of non‐strictly biologically realistic aspects (e.g. ‘all‐to‐all’ connectivity, or back‐propagation learning; Rumelhart *et al*., 1986) would likely lead to a more efficient – from an engineering point of view – system (i.e. exhibiting better learning performance or increased memory capacity), the adoption of any such non‐biological features would undermine the neuroscientific relevance of the model, preventing us from using the present simulation results as a basis to make claims about corresponding brain processes, in focus here.

Previous simulations have shown that, subsequent to the repeated concomitant presentation of activation patterns to (possibly indirectly) linked model areas, networks including the above range of neurobiologically realistic features give rise to the formation of distributed associative circuits (Garagnani *et al*., 2007, 2008, 2009b) corresponding to what Hebb once postulated and labelled ‘cell assemblies’ (CAs; Hebb, 1949). CAs can be defined structurally as sets of nerve cells that are ‘… more strongly connected to each other than to other neurons’ (Braitenberg, 1978a). They constitute ‘memory circuits’ that emerge as a result of correlational learning mechanisms and bind together sets of neurons that are frequently co‐active (Hebb, 1949; Braitenberg, 1978a; Palm, 1982). Once developed, CAs behave as coherent functional units with two quasi‐stable states (‘on’ and ‘off’; Garagnani *et al*., 2007, 2008, 2009b; Pulvermüller & Garagnani, 2014). Cortical CAs whose formation is driven by correlated sensory and motor information are also called action‐perception circuits (Pulvermüller & Fadiga, 2010). Here, we simulated the spontaneous formation of CAs linking symbols (word forms) to aspects of their meaning manifest in information about objects or actions they refer to.

### Network structure and connectivity of the simulated brain areas

The original model of the language cortex simulated six left‐perisylvian areas (three in the inferior fronto‐central cortex and three in the superior‐temporal auditory system; Fig. 1A); here this model is augmented with six new areas (and relevant connections between them) having a role in transferring and processing semantically relevant information. Because these ‘semantic’ areas are outside the perisylvian (language) cortex, in the remainder of this article they are referred to as ‘extrasylvian’ areas. The extrasylvian areas include dorsolateral fronto‐central motor, premotor and prefrontal cortices (M1_*L*_, PM_*L*_, PF_*L*_), and three areas constituting the ventral occipito‐temporal visual ‘what’ stream (V1, TO, AT). Thus, within both peri‐ and extra‐sylvian systems, we distinguished between a ‘dorsal stream’ section, situated in the frontocentral cortex (depicted in different shades of red/yellow) and a ‘ventral stream’ section, in the temporal and occipital cortex (in shades of blue/green) were distinguished between.

Neuroanatomical evidence shows that adjacent cortical areas tend to be connected with each other through next‐neighbour between‐area links (Pandya & Yeterian, 1985; Young *et al*., 1994, 1995). These exist within each triplet of areas of the four systems modelled, that is, amongst: (1) inferior frontal areas PF_*i*_ – PM_*i*_ – M1_*i*_; (2) superior‐lateral frontal areas PF_*L*_ – PM_*L*_ – M1_*L*_ (see also Arikuni *et al*., 1988; Lu *et al*., 1994; Dum & Strick, 2002, 2005); (3) superior and lateral auditory areas A1 – AB – PB (Pandya, 1995; Kaas & Hackett, 2000; Rauschecker & Tian, 2000); and (4) inferior temporo‐occipital areas V1 – TO – AT (Distler *et al*., 1993; Nakamura *et al*., 1993).

Evidence also indicates the presence of long‐distance cortico‐cortical links (see purple arrows in Fig. 1B) connecting areas distant from each other. Amongst the long‐distance links within the fronto‐temporo‐occipital cortex, only the well‐documented mutual and reciprocal connections between anterior temporal, superior parabelt, and inferior, and posterior‐superior‐lateral prefrontal areas were implemented. The connections between anterior (and middle), inferior, and posterior‐superior temporal cortex (areas AT, PB in Fig. 1B) and inferior prefrontal (and premotor) cortex (PF_*i*_) are realised by the arcuate and uncinate fascicles (Makris *et al*., 1999; Romanski *et al*., 1999b; Petrides & Pandya, 2001, 2009; Catani *et al*., 2005; Parker *et al*., 2005; Romanski, 2007; Rilling *et al*., 2008; Makris & Pandya, 2009; Petrides *et al*., 2012; Rilling, 2014). Dorsolateral prefrontal (and premotor) cortex (PF_*L*_) is reciprocally linked to anterior and inferior temporal regions (AT; Pandya & Barnes, 1987; Ungerleider *et al*., 1989; Webster *et al*., 1994), as well as to the superior temporal cortex (PB) via the extreme capsule (Pandya & Barnes, 1987; Romanski *et al*., 1999a,b; Schmahmann *et al*., 2007; Dick *et al*., 2014).

### Simulating semantic symbol grounding

At the onset of learning the network was in a ‘naïve’ state, i.e. one in which all between‐ and within‐area synaptic links connecting single cells were established at random, as were their synaptic efficacies (weights). Word learning and semantic grounding were then simulated by means of repeated ‘learning trials’, involving concomitant stimulation of primary areas of the network, as described below.

As each spoken word form is characterized by an articulatory motor schema and an acoustic schema, each learning trial entailed concurrent stimulation of inferior‐frontal primary motor and superior‐temporal primary auditory areas (perisylvian primary areas M1_*i*_ and A1 in Fig. 1). As some words are typically used to speak about visually perceivable objects and one typical learning situation for such words is the use of the word while the referent object is present (Vouloumanos & Werker, 2009; Barros‐Loscertales *et al*., 2012), learning of object‐related words was simulated by concurrent stimulation of both perisylvian primary areas plus visual cortex (V1). Similarly, learning aspects of action‐related word meaning involved simultaneous activation of primary perisylvian and lateral motor areas (M1_*L*_); this was meant to simulate a situation in which action words are used when the learning child performs the corresponding action (Tomasello & Kruger, 1992).

The learning of six object‐ and six action‐related words was simulated, each by concurrent stimulation of three of the four primary areas, with one specific sensorimotor pattern of neuronal activation for each word. Each sensorimotor pattern consisted of a set of 19 cells per primary area (57 cells in total), randomly selected amongst the 25‐by‐25 cells forming one area (about 3% of cells). Each of the 12 sensorimotor patterns was presented in 3000 learning trials, resulting in a total of 36 000 (randomly ordered) trials. (This number was chosen empirically, on the basis of previous simulations obtained with six‐area architectures; such studies showed the presence of cell‐assembly circuits already after 50–100 trials, and no substantial changes occurring between 1000 and 2000 presentations; Garagnani *et al*., 2009b. Here the training was extended to 3000 presentations per pattern, as the network had to develop CA circuits spanning nine instead of six interconnected areas, linking three patterns instead of just two.) Therefore, the same ‘core’ of neurons were stimulated during each presentation of a given pattern; however, white noise was always present and overlaid the sensorimotor input patterns, so as to account for a degree of variability in the physical features of word forms and semantically relevant objects and actions. Each learning trial lasted 16 simulation‐time steps (equivalent to approximately 300 ms) and was followed by a resting interval of variable duration during which no input was provided until activity had returned to baseline. A new trial started as soon as the global inhibition levels in both areas PF_*i*_ and PB dropped below a pre‐specified threshold (0.65 in the present simulations). As object words are less informative about motor activities than action words, and the latter typically convey less visual information than the former, a static noise pattern was presented to the ‘non‐partaking’ primary area during training. Hence, in each action‐ (object‐) related word learning trial, area V1 (M1_*L*_) was stimulated with a different random pattern of 19 cells. This was intended to mimic the presumably larger variability of the above relationships (or, equivalently, the lower degrees of correlation).

Thirteen different instances of randomly initialized networks having the architecture described above were implemented and subjected to the same learning procedure, each instance being trained with a different set of 12 sensorimotor patterns. As both action and object word meanings may be acquired even if congruent visual or motor information is not consistently present in each episode of learning, the ability of the model to develop word circuits when the (modality‐specific) semantic component of the input pattern is provided only in a fraction of the learning trials was also investigated. To do this, the above set of simulations was repeated under three different conditions, in which the fraction of learning trials containing semantic input varied from the initial 100% to 75, 66.7 and 50% (these fractions are the result of replacing the pattern normally presented as input to V1 or M1_*L*_ with a random, static one once every four, three, and every other trial, respectively). Again, 13 different instances of randomly initialized networks were trained in each condition.

### Data analysis

As further explained in the Results below, the training led to the emergence of CA circuits in the network, that is, sub‐networks of strongly and reciprocally connected cells linking together specific sensorimotor patterns in primary areas by way of cells in intermediary areas. The following procedure was applied to define and quantify the emerging CAs.

After training, the neurons forming each of the 12 CAs across the different network areas were identified. To this end, the response of all 7500 excitatory cells to each of the 12 word‐form patterns was recorded. More precisely, the time‐averaged output (firing rate) of each excitatory cell was estimated over the 15 simulation steps following a single test‐presentation of the auditory and articulatory patterns of a learnt word form (no semantic input was provided). An excitatory cell was then considered a member of the CA for pattern *w* if and only if its (estimated) time‐averaged response to *w* reached a given threshold θ. The threshold θ was area‐ and cell‐assembly specific, and defined as a fraction γ of the maximal single‐cell response in that area to pattern *w*. More formally,

$$\theta =\theta_{A}\left(w\right)=\gamma \max_{x\in A}\bar{O{\left(x,t\right)}_{w}}$$

where $\bar{O{\left(x,t\right)}_{w}}$ is the estimated time‐averaged response of a cell *x* in area A to word pattern *w*, and γ ∈ [0, 1] is a constant (function *O(x,t)* is defined in Appendix A). For the statistical analysis (see below) γ* *= 0.50 was used; this value was chosen on the basis of simulation results obtained with the present and previous networks (Garagnani *et al*., 2008, 2009b). Following standard definitions in the literature on auto‐associative memories (Braitenberg, 1978a; Palm, 1990), only excitatory cells were considered to be part of an assembly.

This definition yields specific numbers of cells per area for each CA that emerged during learning. For each of the 13 network instances, per‐area CA‐cell numbers were averaged over the six object‐related words and over the six action‐related words. To statistically test for possible differences in CA topographies between word types, per‐area numbers of CA cells obtained from the 13 network instances were submitted to repeated‐measure analyses of variance (anovas). A four‐way anova on the data from all 12 areas was performed, with the factors ‘extra/perisylvian (ExtraPeri)’ (two levels: perisylvian = {A1, AB, PB, M1_*i*_, PM_*i*_, PF_*i*_}; extrasylvian = {V1, TO, AT, M1_*L*_, PM_*L*_, PF_*L*_}), ‘frontotemporal (FrontoTemp)’ (two levels: frontal areas = {M1_*L*_, PM_*L*_, PF_*L*_, M1_*i*_, PM_*i*_, PF_*i*_}, temporal areas = {A1, AB, PB, V1, TO, AT}), ‘modality‐specific vs. multimodal (ModSpecificity)’ (three levels: primary unimodal = {A1, V1, M1_*L*_, M1_*i*_}, secondary mesomodal = {TO, AB, PM_*L*_, PM_*I*_} and multimodal = {PB, AT, PF_*L*_, PF_*i*_}), and ‘WordType’ (two levels: object‐, action‐related). Furthermore, two separate three‐way anovas were run on the data from the six extrasylvian and six perisylvian areas (factors ‘FrontoTemp’, ‘ModSpecificity’ and ‘WordType’, as above).

## Results

**Figure 2:**
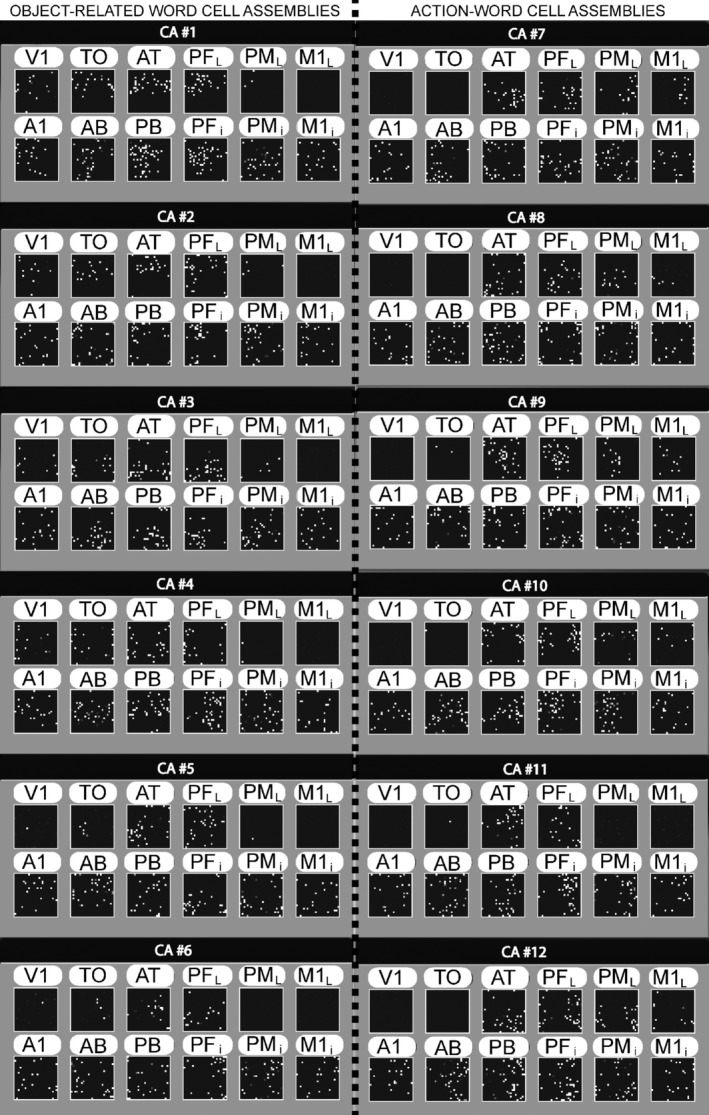
Distributions of cell assembly (CA) circuits emerging in the model during simulation of word learning in the semantic context of visual perceptions (left‐hand side) and actions (right‐hand side). Results from a single instance of the network architecture presented in Fig. 1B are shown. Each set of 12 squares depicts the distribution of ‘cells’ of one specific CA across the 12 network areas. Each white pixel in a square indexes one CA cell. CAs for object‐related words extend into higher and primary visual cortex (V1, TO, but not M1_*L*_), linking information about spoken word forms (perisylvian pattern) with information from the visual modality (neural pattern in V1). Network correlates of action‐related words extend into lateral motor cortex (M1_*L*_, PM_*L*_, but not V1), thus semantically grounding words in information about actions. Note that, on one occasion, this specific network instance failed to learn the association between spoken word‐form and corresponding meaning (see word‐related CA #11, which does not reach into area M1_*L*_).

Figure 2 depicts representative examples of CA topographies emerged during simulated learning of twelve words semantically grounded in either object (left) or action (right) information. CA circuits of the two semantic types exhibit similar distributions over the perisylvian cortex, with the highest CA‐cell densities emerging in the multimodal areas PF_*i*_ and PB. By contrast, extrasylvian motor and visual areas appear to exhibit a double dissociation: action‐related word learning yields CAs including cells in lateral premotor and even primary motor cortex of the model, but weakly developed or virtually absent in visual areas TO and V1. Conversely, learning words with an object‐related meaning seems to produce circuits biased towards the visual system. Finally, CA circuits for both action‐ and object‐related words appear to include comparably large cell densities in multimodal extrasylvian areas AT and PF_*L*_.

**Figure 3:**
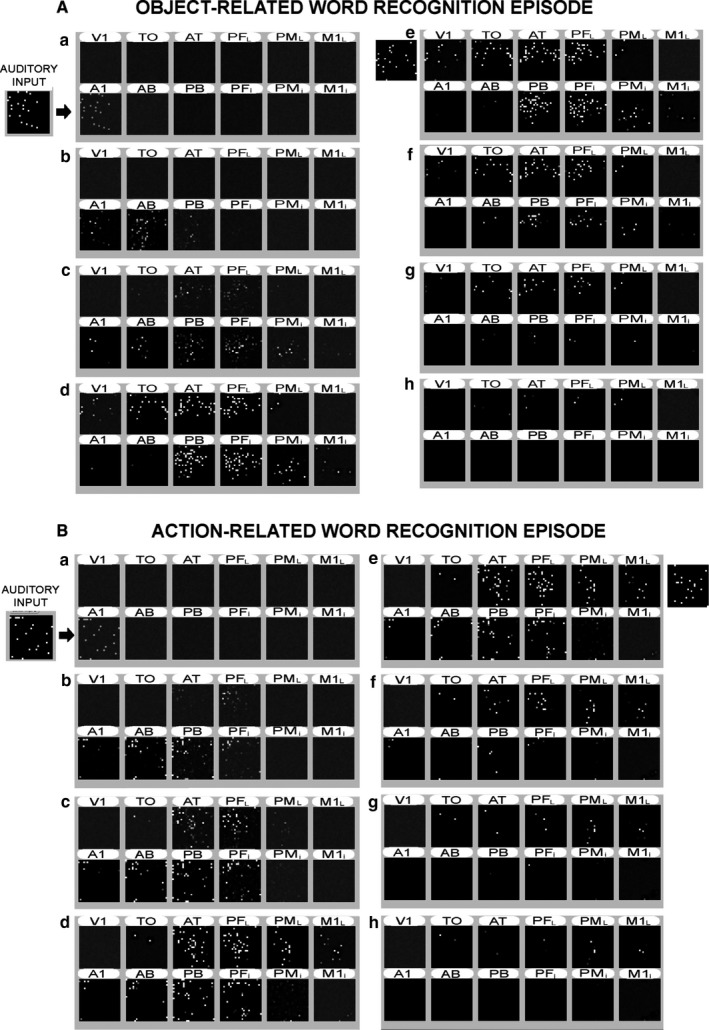
Activation spreading in the network during simulated word recognition. Representative snapshots of network responses to stimulation of A1 with the ‘auditory’ component of a learned object‐related (A) and action‐related (B) word (see CA #1 and CA #9 in Fig. 2, respectively); each set of 12 ‘squares’ captures the network's instantaneous activity. Cell‐activity levels are indicated by brightness of pixels; letters indicate chronological order (not simulation time‐steps). For ease of visual comparison, the original sensory and motor patterns that the network was trained with are reported in the top‐right snapshots (frame ‘e’) of both (A) and (B). The pattern reconstruction is partial and strongly involves visual areas in (A) and motor areas in (B). See main text for details.

Figure 3 illustrates examples of CA‐circuit activation dynamics during two simulated word‐recognition episodes. Here, only the ‘auditory’ component (area A1) of a learnt sensorimotor word‐pattern was presented as input to the network, causing the ‘ignition’ of the CA circuit that had emerged for that specific word. As visible in the figure, this is a near‐simultaneous activation process that involves several areas of the network. In line with the differential topographies shown in Fig. 2, object‐related word recognition activity extends well into areas V1 and TO, but not to M1_*L*_, and only marginally to PM_*L*_, whereas action‐word CA ignition reaches M1_*L*_ and PM_*L*_, but not V1, and only marginally TO. Importantly, as CAs for words of either category heavily draw upon the four ‘central’ (perisylvian and extrasylvian) multimodal hub areas, simulated object‐ (Fig. 3A) and action‐related (Fig. 3B) word‐recognition processes appear to induce comparable levels of activity there.

**Figure 4:**
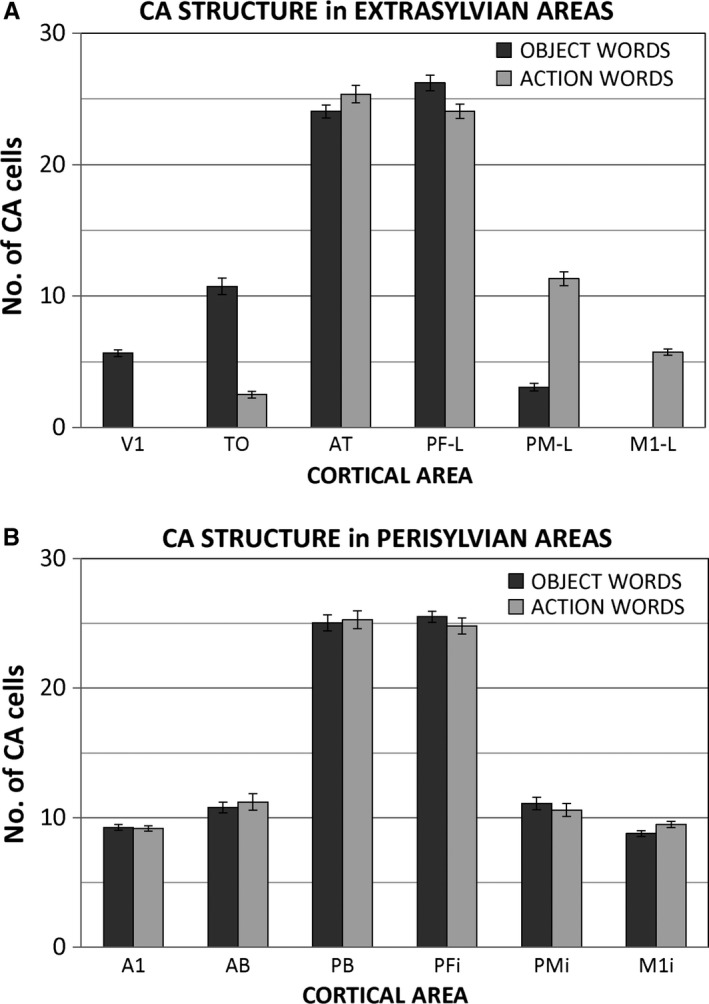
Average distributions of cell assemblies (CAs) emerging in 13 instantiations of the 12‐area network architecture during simulation of word learning in the semantic context of actions and visual perceptions. Bars show average numbers of CA neurons per area (or ‘CA‐neuron densities’) for object‐ (dark grey) and action‐related (light grey) word representations; error bars indicate standard errors over networks. (A) The extrasylvian areas, whose cells can be seen as circuit correlates of word meaning, show a double dissociation, with relatively more strongly developed CAs for object‐ than for action‐related words in primary and secondary visual areas (V1, TO), but stronger CAs for action‐ than for object‐related words in dorsolateral primary motor and pre‐motor cortices (PM_*L*_, M1_*L*_). Note the coexistence of symbolic CA circuits having comparable densities for either semantic category in the multimodal ‘hub’ areas AT and PF_*L*_. (B) Data from the six perisylvian areas, whose cells can be seen as circuit correlates of spoken word forms, do not show category‐specific effects.

The results of the statistical analysis presented in Fig. 4 fully confirmed the above empirical observations. The graphs in Fig. 4A and B plot the number of CA cells per area that emerged with the training, averaged across 13 different network instances (CA cells were identified using the definition given in ‘Data analysis’). The four‐way anova run on the data from all 12 areas revealed a main effect of ModSpecificity (*F*_2,11_ = 2345, *P* < 0.001), with generally more CA cells and therefore higher assembly‐cell density in the multimodal than in the secondary mesomodal (*t*_12_ = 48.9, *P* < 0.001), and in the secondary than in the primary unimodal (*t*_12_ = 14.6, *P* < 0.001) areas; in addition, a highly significant interaction of the factors ExtraPeri, ModSpecificity, FrontoTemp and WordType (*F*_2,11_ = 137.6, *P* < 0.001) emerged, confirming that CA topographies (i.e. the distributions of their neurons over the areas) differed between word types. As the distinction between peri‐ and extra‐sylvian areas had a significant influence here, topographical word‐type effects were further investigated separately for the perisylvian core language areas and the extra‐sylvian ones. The three‐way anova run on the data from the perisylvian areas did not provide strong evidence for word types differences across areas (although the respective interaction of topography with word type approached significance: *F*_2,11_ = 3.05, *P* = 0.066). In contrast, extrasylvian areas revealed a highly significant interaction of the factors FrontoTemp and ModSpecificity with WordType (*F*_2,11_ = 290, *P* < 0.001), showing semantic word‐category differences in CA topographies in ventral temporo‐occipital and dorsolateral frontal areas. There was also a main effect of ModSpecificity in the perisylvian (*F*_2,11_ = 1345, *P* < 0.001) as well as in the extrasylvian (*F*_2,11_ = 2549, *P* < 0.001) areas, analogous to that revealed by the four‐way anova.

Further, the significant topographic differences between the circuits of action‐ and object‐related words in extrasylvian model areas were explored. Bonferroni‐corrected planned comparison tests (for 12 comparisons, critical threshold *P* = 0.0042) confirmed that larger numbers of cells in V1 and TO were part of circuits for object‐related words than for action words (*t*_12_ > 8.7, *P* < 0.001), whereas the opposite applies to M1_*L*_ and PM_*L*_ (*t*_12_ > 8.96, *P* < 0.001). Extrasylvian multimodal model areas AT and PF_*L*_, which serve as main hubs for visual, auditory and motor information, did not show significant differences between CA types after correcting for multiple comparisons (AT: *t*_12_ = 1.92, *P* = 0.079; PF_*L*_: *t*_12_ = 2.88, *P* = 0.014, n.s. after correction). Analogous *post hoc t*‐tests investigating possible semantic category differences in the perisylvian areas (Fig. 4A) were all not significant (*t*_12_ ≤ 1.5, *P* > 0.13 across all six areas).

**Figure 5:**
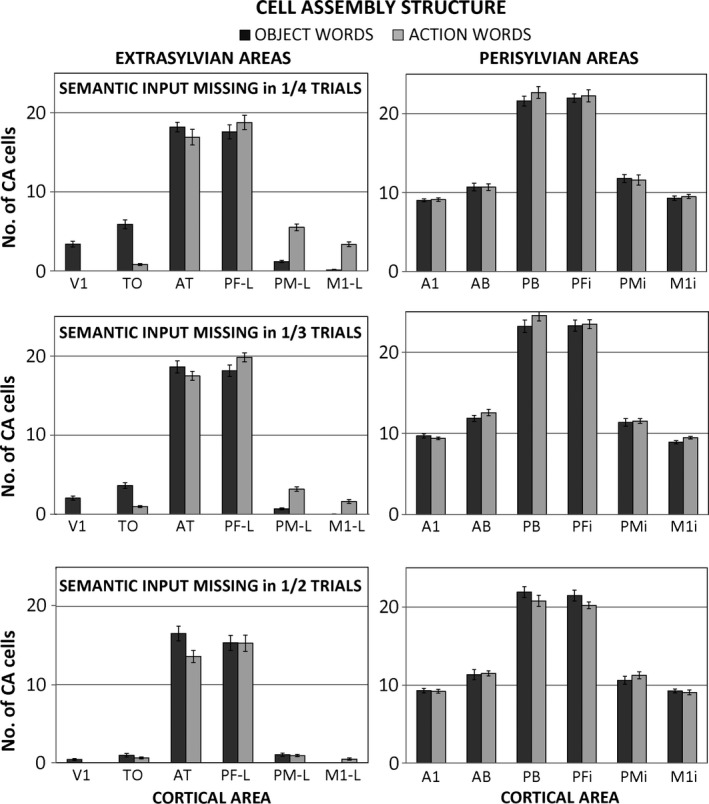
Average distribution of emerging word‐related cell assemblies (CAs) obtained for different amounts of semantic information provided as input during training. Left: data from extrasylvian areas. Note the gradual weakening of CA‐circuits exhibited by both word categories for increasing fractions of trials failing to provide semantic input, ultimately leading (bottom row) to most word circuits not reaching the modality‐specific areas. Right: data from perisylvian areas. CA distributions here are relatively unaffected by the fraction of semantic‐information‐bearing trials (but note that areas PF_*i*_ and PB develop smaller numbers of cells in comparison to data plotted in Fig. 4B).

Last, the impact that the relative amount of congruent visual or motor information provided during word acquisition – or, equivalently, that the variability in the semantic input – had on the emerging topography of the word circuits was examined. Figure 5 plots the resulting object‐ and action‐word CA distributions as a function of the percentage of learning trials in which the semantic pattern normally associated with a word was replaced by a random one. In line with the results obtained when 100% of the trials included semantic input (data plotted in Fig. 4), *post hoc t*‐tests revealed that, for all conditions, word‐category specificity emerged in primary and secondary extrasylvian areas (*t*_12_ > 7.0, *P* < 0.0005, still significant after correcting for 18 multiple comparisons), with the exception of the 50% case, in which the CAs of the two word types did not differ after application of a conservative threshold (*t*_11_ < 3.8, *P* > 0.003 across all areas, n.s. after correction). Moreover, no significant differences between categories emerged in the two extrasylvian hubs AT and PF_*L*_ in any of the conditions (*t*_12_ < 3.2, *P* > 0.009 for all three conditions and two areas, n.s. after correction), or in the perisylvian areas (*t*_12_ < 1.8, *P* > 0.11 across all areas and conditions). These results show that, although inconsistent learning reduces the efficacy of semantic circuit formation, the principal topographical differences indexing category specificity tend to persist.

## Discussion

The present model provides a neurobiological explanation for the emergence of category‐specific effects in modality‐preferential cortices, as well as the consistent activation of multimodal ‘semantic hub’ areas, as observed in the brain during semantic processing. In our neuroanatomically inspired model of perisylvian and extrasylvian fronto‐temporo‐occipital cortex, the distinct category‐specific and category‐general functional behaviours emerged spontaneously in different areas as a consequence of the learning process, in particular, of the simulated semantic grounding of words in information about their referent objects and actions. As discussed below, this is explained by the different topographies of the emerging representations, i.e. the semantic circuits, which, in turn, are determined by the underlying neuroanatomical connectivity structure, Hebbian associative learning mechanisms at work therein, and sensorimotor patterns driving word acquisition and semantic grounding processes.

### Semantic hubs vs. category specificity

In the past, semantic processing has been attributed by some to a symbolic system dedicated to processing conceptual information related to words and symbols (Collins & Loftus, 1975; Potter, 1979; Ellis & Young, 1988). However, neuroimaging and neuropsychological evidence implicating the involvement of several different areas in semantic processing casted doubts on the existence of a single ‘amodal’ meaning centre, suggesting, instead, the presence of several multimodal hubs, located in higher‐association areas of anterior‐inferior‐temporal (Patterson *et al*., 2007), middle‐temporal (Price, 2000), inferior‐parietal (Binder & Desai, 2011) and prefrontal cortex (Bookheimer, 2002; Devlin *et al*., 2003). At the same time, a growing number of neuroimaging and patient studies (e.g., Warrington & Shallice, 1984; Kemmerer *et al*., 2012) lend support to a theory of word meaning grounded in the perception and action systems of the brain (Lakoff & Johnson, 1999; Pulvermüller, 1999; Barsalou, 2008; Pulvermüller & Fadiga, 2010; Binder & Desai, 2011; Glenberg & Gallese, 2012). In particular, evidence confirms the existence of links between word‐form circuits in perisylvian language areas and corresponding semantic information in extrasylvian modality‐preferential sensorimotor ones: action‐related words (such as ‘grasp’ or ‘kick’) spark activity in lateral and superior motor and premotor cortex (Martin *et al*., 1996; Rizzolatti & Craighero, 2004; Aziz‐Zadeh *et al*., 2006; Pulvermüller *et al*., 2009; Kemmerer & Gonzalez‐Castillo, 2010), while semantic processing of visually‐related symbols (such as colour, object or animal words) produces activity in specific visual areas of the ventral temporo‐occipital stream (Damasio *et al*., 1996; Martin *et al*., 1996; Pulvermüller & Hauk, 2006; Martin, 2007; Simmons *et al*., 2007; Carota *et al*., 2012). The evidence for both category‐specific and category‐general semantic areas pleads for a unifying neural model of early language acquisition, able to explain the spontaneous emergence of both in the cortex as a consequence of word learning and semantic grounding. Based on the present simulation results, such a neurobiological account is proposed below.

### A new integrative model of semantic‐category specificity and hubs

First some basic principles of spontaneous CA development, useful in the subsequent explanations, are introduced.

In networks that implement rich auto‐associative connections between neurons along with Hebbian learning, constantly stimulated neurons have the tendency to strengthen their connections to cells they are linked to, so that, with time, larger and larger CAs develop (Doursat & Bienenstock, 2006). However, the spontaneous tendency of stimulated CAs to grow may be offset (or partly limited) by the specific features of a network, such as the density, extent and reciprocity of synaptic projections; in particular, sparse, patchy and topographic (as opposed to ‘all‐to‐all’) connectivity, as implemented here, makes CA growth harder. The presence of uniform white noise (simulating baseline neuronal firing) also has an effect on CA development: as random noise de‐correlates activity between any pair of cells, its net effect is to weaken all synaptic weights in the network, thus generally counteracting CA expansion. Finally, given that CA formation is a consequence of synaptic strengthening driven by Hebbian associative learning, the ‘degree’ of correlation between the patterns of activity that co‐occur in two or more connected areas is a critical factor for determining whether an input‐specific CA circuit linking such patterns will or will not emerge in the network. (Note, in this context, that the presentation of uncorrelated, random activity patterns to the fourth, ‘non‐partaking’ primary area during the network's training specifically hindered the growth of CA circuits into these systems; as discussed below, this was crucial for the development of category‐specific circuit topographies.)

As shown by the model simulations, learning the meaning of action‐ or object‐related words in the context of grounding motor activity or sensory input leads to the formation of input‐specific lexico‐semantic CA circuits in the cortex. These circuits bind the ‘lexical’ representation of a word – which links articulatory and acoustic‐phonological activity patterns in M1_*i*_ and A1, related to spoken word form production and perception, respectively – with a perceptual or a motor schema circuit reaching into primary visual or motor areas (V1 or M1_*L*_; Fig. 1A and B). Because of the absence of direct white‐matter tracts between these modality‐specific primary cortices, however, the word‐related circuits emerge as widely distributed over primary (where the driving activation is present), secondary, and intermediary ‘relay’ areas, through which waves of correlated activity travel during learning. Hence, such multimodal convergence areas and their long‐distance cortico‐cortical connections play a major role in binding phonological/lexical and semantic information, with PB and PF_*L*_ being especially relevant for action‐related words, and PF_*i*_ and AT for object‐related ones (Fig. 1B).

#### Explaining category‐specific effects in modality‐preferential areas

The emergence of distributed CA circuits follows directly from the principles of spontaneous CA growth and the presence of correlated patterns of activity in different sets of primary sensorimotor areas. Depending on the semantic category of the word being learned, different CA circuits exhibiting different distributions across modality‐preferential cortices develop. In particular, CA topographies are biased towards the motor system for action‐related words grounded in motor execution, and towards the visual system for object‐related words grounded in visual perception. Hence, these areas will exhibit category‐specific effects during word processing across different tasks (e.g. word recognition and passive listening) because the associated semantic circuit parts are reactivated along with the spoken word‐form representations. A more precise and detailed explanation follows.

Learning the meaning of object and action words may result from the presence of correlated patterns of activity in two different sets of three primary sensorimotor areas: V1, M1_*i*_, A1 for object‐, and M1_*L*_, M1_*i*_, A1 for action‐related words. This leads to the emergence of strongly‐connected distributed word‐related CA circuits joining together the neurons consistently activated by these sensorimotor patterns; however, because of CA growth principles, the emerging circuits do not extend to areas where neural activity exhibits a low degree of correlation with such patterns, i.e. primary hand‐motor area (M1_*L*_) for object‐, and primary visual cortex (V1) for action‐related words (during training these areas were stimulated with a different random pattern in each learning trial; see Materials and methods). Therefore, in primary and secondary visual areas (V1, TO), densities of object‐related word cells become larger than action‐related ones; conversely, CA neuron densities in motor areas (M1_*L*_, PM_*L*_) are higher for action‐related words than for object‐related ones (Fig. 4B).

It should be clarified here that presenting random‐noise patterns to the not‐directly‐stimulated modality system during training (i.e. to area V1 for action words, and to M1_*L*_ for object words) was necessary to prevent the spontaneous extension of all semantic circuits into both motor and perceptual areas. In fact, in a separate set of simulations six network instances were trained without presenting such noisy patterns; the results showed that CAs extended further into the non‐partaking, ‘silent’ arm of the network, eventually producing a paradoxical distribution in which action word‐circuits reached also into primary visual areas, and object CAs into motor ones. This observation confirms the fundamental role of neuronal noise in preventing excessive CA growth (Doursat & Bienenstock, 2006), and suggests its relevance to semantic grounding processes.

#### Explaining category‐general behaviour of multimodal semantic hubs

The main observation here is that CA circuits for words from different semantic categories co‐exist within the same semantic hub, exhibiting comparable strength (number of cells) there. Thus, cortical hubs will show similar levels of activation during recognition/comprehension of items from any of these categories. In other words, convergence areas behave like ‘multiple demand’, or category‐independent systems because they house CAs for symbols of all semantic types. The cortical mechanisms that, in the present architecture, lead to this result are illustrated below.

First note that the four multimodal convergence areas (AT, PF_*L*_, PB, PF_*i*_) are directly connected with each other (Fig. 1B). Due to the repeated concomitant stimulation of three primary cortices with correlated sensorimotor patterns, CA circuits develop in three of these four hubs areas (including PB and PF_*i*_, plus AT for object‐ and PF_*L*_ for action‐related words). The fourth hub, although not on the pathway connecting the three relevant primary cortices, is reciprocally linked with two other multimodal hubs and therefore receives substantial input during semantic learning. As in the presence of adequate conditions constantly stimulated CAs extend to adjacent areas (see above), the emerging symbolic circuits spontaneously grow into the fourth semantic hub. As word‐related CA circuits of both semantic categories extend into both multimodal areas AT and PF_*L*_ (Fig. 4B), neurons in both will be active during semantic processing of symbols from either category (Fig. 3).

It should be noted that CA circuits are stronger (i.e. contain more CA cells) in hub areas, which ‘interface’ between the different modality systems, than in modality preferential ones (Fig. 4). This appears to be a general feature of the present type of architecture (Garagnani *et al*., 2008; Garagnani & Pulvermüller, 2013; Pulvermüller & Garagnani, 2014), in which ‘central’ multimodal areas exhibit on average the highest numbers of synaptic links to other areas (in terms of connectivity, the highest ‘degree’; van den Heuvel & Sporns, 2013). To see this, refer to the connections depicted as arrows in Fig. 1B: primary cortices are linked with only one area, secondary ones with two, while all multimodal areas have three incoming/outgoing arrows. Cells with larger numbers of incoming/outgoing projections have a generally higher probability of being (randomly) linked to cells that happen to exhibit a correlated pattern of activity; in the presence of Hebbian learning, this implies a higher probability to become part of a CA (Garagnani *et al*., 2009b). Furthermore, because these areas are the point of convergence of different streams of sensorimotor input, they are likely to receive more excitatory input than the modality‐preferential ones, and more active cells are more likely to undergo synaptic changes [see Eqn A5 in Appendix A].

The spreading of activity from the phonological/lexical (perisylvian) part of the CA to the extrasylvian hubs, and from there to the secondary and primary areas of modality preferential systems, is taken here to be a model correlate of the cortical processes underlying semantic understanding. In this sense, the above results suggest that, while modality‐preferential cortices certainly contribute to word meaning acquisition and comprehension (as they enable encoding and recollection of item‐specific sensorimotor information), the majority of ‘semantic neurons’ emerge in convergence zones, where phonological and semantic word‐circuit parts are bonded. Due to their role as structural‐neuroanatomic connection hubs and integration points of multimodal activity, such areas end up housing word‐related CAs of all different types, and hence become involved in the processing of items of all semantic categories. Thus, it is proposed here that the strong activations that multimodal hub areas often exhibit during semantic processing are the result of the presence of large numbers of neurons of all semantic circuit types there (which, in turn, follows directly from neurobiological principles and connectivity structure).

One might speculate that the convergence zones may also be the locus where information about different specific referent exemplars is integrated into a single, conceptual representation (Patterson *et al*., 2007). However, as basic visual features of objects falling under a referential term (or movement trajectories of different action types) show surprising similarity across instantiations, a role of secondary and even primary modality‐preferential areas in such integration appears possible. [Note that two synaptic steps – needed to compute more complex logical operations such as either‐or relationships (Kleene, 1956; McClelland and Rumelhart, 1986) – are often necessary to categorize different exemplars/semantic features under the same concept; in the current model, such integration would only be possible in higher‐order areas. However, here only one excitatory layer per area was implemented; this is a modelling simplification, as several neuronal steps are actually possible within the six cortical layers of each local neuronal cluster (Braitenberg & Schüz, 1998). With several synaptic steps in each area, either‐or and similarly complex computational integration would be possible even in primary fields.] Previous simulations indeed demonstrated the ability of a similar (six‐area) architecture to spontaneously ‘merge’ different overlapping CA circuits into a single one; this is because correlation learning tends to omit variable information and strengthen common features. This phenomenon, however, depended on the amount of overlap between the input patterns, as well as on the specific learning rule adopted (Garagnani *et al*., 2007, 2009b), factors that were not the focus of the present investigation.

It should be emphasized that the ability of the model to develop strong symbolic CA circuits linking up phonological (perisylvian) and semantic (extrasylvian) circuit parts persists if the semantic pattern is absent in up to 33% of the learning trials (Fig. 5). With 50% of missing trials, the category‐specific nature of the emerging circuits only persisted as a trend, which fell victim to conservative correction for repeated statistical testing. This result demonstrates not only the network's robustness to acquire the meaning of an action or object word even when congruent visual or motor activation is missing (as it often happens in reality), but also the tolerance of the architecture to an increase in the variability of the semantic input (in the simulations, decreases in the fraction of semantic‐information‐bearing trials were reflected by corresponding increases in the proportion of random vs. meaningful patterns presented to the relevant primary area in association with each individual word).

**Figure 6:**
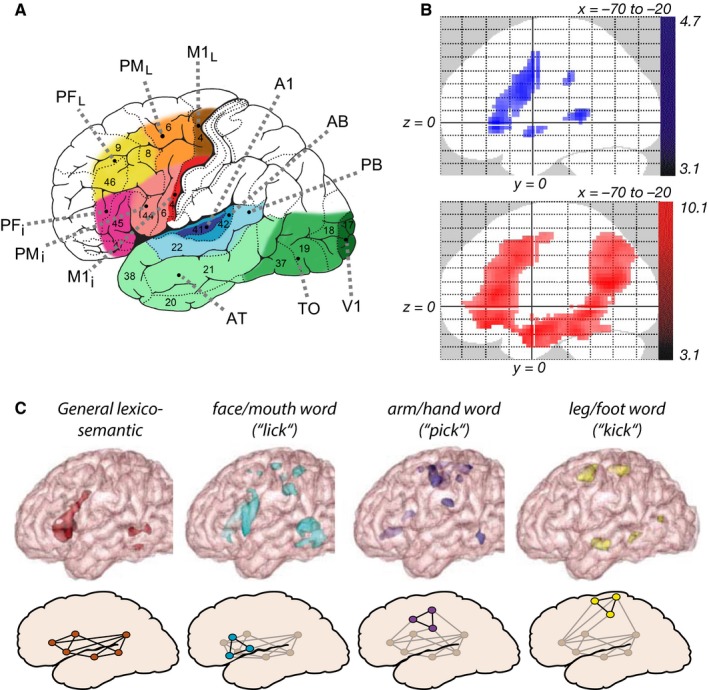
Comparison between simulated areas/processes and foci of real cortical activations as observed during language processing tasks. (A) The postulated mapping of model areas onto specific cortical regions (repeated from Fig. 1A for ease of comparison). Note the ‘nesting’ of the smaller perisylvian lexical/phonological areas (A1, AB, PB, PF_*i*_, PM_*i*_ and M1_*i*_) within the larger extrasylvian semantic one (V1, TO, AT, PF_*L*_, PM_*L*_ and M1_*L*_). (B) Cortical areas activated by ‘phonological’ (top – repetition of pseudowords compared with words) and semantic‐comprehension (bottom – listening to normal sentences compared with meaningless pseudo sentences) tasks (adapted from Saur *et al*., 2008, their Fig. 1, © 2008 National Academy of Sciences, USA). In the repetition task, stimuli consisted of 60 German words and 60 meaningless pseudowords. In the comprehension task, stimuli consisted of 90 well‐formed German sentences [e.g. ‘der pilot fliegt das flugzeug’ (the pilot flies the aeroplane)] and 90 meaningless pseudo sentences (e.g. ‘ren simot plieft mas kugireug’). Note the strong activation of the model's category‐general semantic hubs (PF_*L*_, AT) along with other extrasylvian areas produced by the comprehension task (red areas) but not by the repetition task (blue areas), which, instead, activates mostly perisylvian areas. Also note the approximate nesting of red areas within blue ones. (Parietal areas were not modelled in the present study.) Activations are overlaid as maximum intensity projections (*x*, 70–20) on a canonical brain; statistical threshold was set at *P *< 0.001, uncorrected. (C) Results of cluster analysis (from Pulvermüller *et al*., 2009) revealing activation clusters common to different word types (leftmost column) and activations produced by different semantic word categories (other three columns). Stimuli consisted of five matched sets of 50 English words from five semantic categories: arm/hand‐, face/mouth‐ and foot/leg‐related action words, plus form‐ and colour‐related words. Subjects were instructed to attend to all stimuli flashed on the screen and to silently read the words. The analysis contrasted activation patterns elicited by individual word categories (each tested against a control condition of matched meaningless symbol strings) with each other and with those activations shared by combinations of semantic categories. While general lexico‐semantic circuits shared by different word types appear circumscribed to multimodal hub PF_*i*_, clusters produced by action‐related words extend (somatotopically) to modality‐preferential areas of the model – in particular, note the precise overlap between arm/hand activation and category‐specific model areas PM_*L*_/M1_*L*_ (adapted from Pulvermüller *et al*., 2013, with permission).

As mentioned in ‘Semantic hubs vs. category specificity’, one of the main contributions of this model is to explain the emergence and topography of areas for category‐specific and category‐general semantic processing. If the mapping of model‐ to brain‐areas provided in Fig. 1A is, in spite of its coarseness and simplified structure, appropriate in relevant aspects, the topography of the emerging word‐circuit distributions (Fig. 4) and the corresponding activation patterns observed during simulated word recognition (Fig. 3) should match, to a degree, the patterns of brain activity observed experimentally during semantic processing. To enable a direct comparison of simulated and real brain responses, and assess the level of spatial accuracy of the mapping proposed, Fig. 6 reports the cortical areas identified by the model along with examples of phonological, category‐general and category‐specific semantic activations as revealed by recent functional magnetic resonance imaging studies. In particular, Fig. 6B (adapted from Saur *et al*., 2008) shows the different brain systems activated by two different language tasks, thought to indicate phonological (top) and semantic (bottom) brain processes. Note that the areas found active in the ‘phonological’ contrast (repetition of pseudowords compared with words) are mainly perisylvian, whereas the ‘semantic’ contrast (listening to normal sentences compared with meaningless pseudo sentences) reveals some prefrontal and superior temporal activity along with dorsolateral prefrontal and anterior to middle temporal activity (PF_*L*_, AT), also reaching into the parietal cortex. These systems exhibit a substantial degree of overlap with the perisylvian (phonological/lexical) and extrasylvian (semantic) systems of the model, respectively (see caption for details). Figure 6C (adapted from Pulvermüller *et al*., 2009) reports patterns of activation induced by the processing of: (1) different word types (leftmost column); and (2) words from three specific action‐related semantic categories (columns 2–4). Action‐related words specifically activate modality‐preferential superior and lateral motor areas; note, in particular, that the major cluster activated by arm/hand words (column 3) corresponds to two model areas (PM_*L*_, M1_*L*_) where neuron densities of action‐related symbolic circuits were enhanced in a category‐specific manner (see Fig. 4A).

### Model limitations, future extensions and predictions

Like any model, the present neural architecture makes a number of simplifying assumptions, and is therefore limited in several ways. Firstly, the connectivity realised includes just a subset of the links known to exist between the relevant cortices. In fact, the neuroanatomy of both the auditory and visual (as well as prefrontal) cortices is much more complex than that realised here (Felleman & Van Essen, 1991; Kaas & Hackett, 2000; Petrides & Pandya, 2001, 2009; Vincent *et al*., 2007; Rauschecker & Scott, 2009; for a recent discussion on perisylvian connectivity, see also Garagnani & Pulvermüller, 2013). It is important to emphasize, however, that there is good experimental evidence for the existence of all links that the model implements (see ‘Network structure and connectivity of the simulated brain areas’). The choice of deploying a network implementing only a minimal set of links can be justified on the basis of practical as well as methodological considerations: besides the need to keep simulation time within acceptable ranges, starting with a ‘light’ network structure is motivated by the observation that the introduction of more connections should preserve any CA circuits already emerging in the basic version, with the possible additional effect of making such representations more strongly connected and therefore more stable. This hypothesis is supported by previous simulations (Garagnani *et al*., 2008; Pulvermüller & Garagnani, 2014). Nevertheless, while an ‘Occam's razor’ strategy is appropriate for a proof‐of‐concept study like the present one, some of the results obtained here may be the consequence of such simplification; further simulations are necessary to investigate emergence, distribution and dynamics of CA circuits in networks implementing richer connectivity and additional areas (see below).

Secondly, in real situations, learning the meaning of object and action words might involve the concurrent presence of correlated activity in motor as well as visual areas (Pulvermüller, 1999; Pulvermüller & Fadiga, 2010). For example, when acquiring the meaning of the word ‘grasp’ while performing grasping actions, correlated activity is likely present not only in language and motor systems but also in the ventral visual ‘what’ stream (e.g. if the same object is being repeatedly grasped; Ungerleider & Mishkin, 1982; Mishkin *et al*., 1983; Ungerleider & Haxby, 1994), as well as in the dorsal parieto‐occipital visual ‘where’ stream (Jeannerod *et al*., 1995; Arbib, 1997; Kiefer & Spitzer, 2001), not modelled here. The main target of the present study – to differentiate and explain the spontaneous emergence of category‐specific and more general semantic mechanisms in the brain – motivated a focus on the modality that provides the sensorimotor features most relevant for semantic learning. In this sense, it may be justified to focus on motor features of actions and visual features of objects: these can be seen as relatively constant, whereas the visual features of actions can be quite variable (think of the many different objects that can be grasped with a power grip), as can the action aspects of many objects. Still, some items, especially foods and tools, have both prototypical visual features and very specific motor affordances, so that semantic learning should, in their case, include both visual and motor patterns (Warrington & McCarthy, 1987; Martin, 2007). Thus, an important direction for future extensions of the model consists in the addition of parietal areas and the implementation of sensorimotor information that can reflect both phonological and semantic features of words, symbols, actions and objects.

Although this study aimed to explain aspects of the empirically documented role of given brain areas in category‐specific and ‐general semantic processing, the model was not designed to fit any particular set of behavioural data related to word comprehension. (Note, however, that previous simulations with a similar architecture were used to predict and explain specific brain activation patterns reflecting the processing of lexical information or the role of attention in language processing; Garagnani *et al*., 2008, 2009a; Garagnani & Pulvermüller, 2011). In spite of this, the current neural‐network model may already be capable to replicate and explain additional behavioural results, for example concerning priming effects between semantically related stimuli and symbols. There are at least two ways in which the semantic relationship between words and concepts could be implemented here: first, by overlap of sensorimotor patterns (as for the concepts ‘CUP’ and ‘GLASS’, where referents have visual features in common; Barsalou, 1999); second, by combination, i.e. co‐activation of different CA circuits. Stimulation overlap leads to overlap in the CA circuits, providing a putative basis for semantic feature overlap, as assumed in semantic feature theories (Katz & Fodor, 1963). Co‐activation of CAs will, in the presence of Hebbian learning, strengthen existing links between them, leading to their association; this could capture basic combinatorial semantic relations between words, as assumed by distributional semantic theories (Collins & Loftus, 1975; Landauer & Dumais, 1997).

Another simplifying assumption of the model that begs for further extensions consists of simulating one action (and, likewise, object) as one static motor (visual) activation pattern, whereas, realistically, a fine‐grained structure of more or less prototypical variants might have been desirable (note, however, that the presence of random noise in all areas, overlaid to the input patterns during learning, captures, to some extent, this variability). Similarly, the acoustic‐phonological and articulatory‐phonological patterns presented as input to the A1 and M1_*i*_ areas were fixed, and did not mimic the natural variation in sound categories observed in real speech. Such variability could be introduced in the simulations by replacing a single input pattern with a set of partly‐overlapping instances, obtained by random variation of the same prototype. As mentioned earlier, partly overlapping input patterns may lead to the emergence of a ‘joint’ CA circuit, merging the different CAs into a single one. Whether this will happen, however, depends not only on the degree of overlap, but also on the synaptic plasticity rule adopted, as well as other network parameters, including noise level and density and width of cortico‐cortical projections (see also Garagnani *et al*., 2008, 2009b for a discussion). Further simulation studies systematically manipulating features of the input patterns are needed to assess more thoroughly the model's ability to handle additional variability and account for key linguistic effects related to semantic processing.

It should be noted that while standard psycholinguistic models define *a priori* different layers as having different linguistic functions (phonological, lexical, semantic; Dell *et al*., 1999), in the present simulations phonetic/phonological and semantic‐referential information is co‐presented to primary areas, and lexico‐semantic circuits emerge as a result of learning. Therefore, the network develops internal lexico‐semantic representations spontaneously, according to neurobiological principles known to govern brain function. This approach is seen as explanatory and an improvement upon *a priori* defining the function of the different network's layers. The following mapping between linguistic levels and network parts exists or emerges here: articulatory and acoustic phonetic/phonological features are implemented in areas M1_*i*_ and A1, semantic features in M1_*L*_ and V1, and lexico‐semantic symbolic representations are distributed circuits spanning the entire network.

The results of the simulations enable us to make critical predictions about (and/or explain) the different extents of involvement of relevant multimodal, secondary and primary cortices during acquisition and processing of novel object‐ or action‐related words; these predictions can be tested in (and inspire the implementation of) novel neuroimaging experiments. For example, the model results lead us to predict that none of the perisylvian areas should show significant category‐specific effects (Fig. 4A), i.e. object‐ and action‐related word circuits should not exhibit differences in their perisylvian distribution. This is not a trivial consequence of the fact that the two word types did not exhibit systematic differences in their auditory‐articulatory forms; in fact, the training process involved asymmetric stimulation of the network, with triplets of correlated patterns being presented to three of the four primary areas of the model (see ‘Simulating semantic symbol grounding’) – indeed, this asymmetry drives the resulting CA‐cell distribution in the extrasylvian areas exhibited by the two semantic categories. In view of this, one might have expected the presence of asymmetries in the perisylvian distributions (as well as in extrasylvian ones). Second, the network predicts the emergence of more ‘semantic’ CA neurons in secondary (extrasylvian) areas than in primary ones (PM_*L*_ > M1_*L*_ and TO > V1). This unexpected result can be explained in terms of CA growth principles, whereby the larger numbers of CA cells that multimodal hubs develop (for the reasons discussed earlier) lead to the recruitment of more CA cells in the nearby (directly connected) secondary areas than in the non‐adjacent primary ones. Third, according to the present modelling results, we would not predict category‐specific activity in semantic hubs. Category effects should therefore only emerge where hubs interface with the ‘secondary’ semantic areas delineated in the model. Further precise predictions about the spreading and time course of semantic activation can be made, for example for word recognition tasks (Fig. 3), and related to empirical results (Moseley *et al*., 2013; Shtyrov *et al*., 2014). It should be emphasized, however, that most previous experimental works showing specificity of cortical areas to semantic categories used words from natural languages, where the way these items have been learned cannot be adequately controlled for. In order to properly test the predictions resulting from the present model, word learning experiments are needed, in which neuroimaging techniques with high spatial/temporal resolution are used to reveal emergence, dynamics and distribution of CA circuits for newly‐learned action‐ and object‐related words. The prediction that action‐semantic circuits reach into (and therefore their activation should spark) the premotor and primary motor cortex is in line with a number of experimental studies (Hauk *et al*., 2004; Tettamanti *et al*., 2005; Kemmerer *et al*., 2012; Shtyrov *et al*., 2014). However, evidence for the activation of the primary visual cortex during object‐related word processing (Martin *et al*., 1996; Pulvermüller *et al*., 1999) is somewhat sparse, as most category‐specific differences have been seen in more anterior temporal cortices. Thus, in future experiments testing the present simulation study's predictions it will be crucial to examine in detail visual cortex activation to specific object‐related symbol categories.

We conclude on a speculative note. Recent comparative neuroimaging studies have confirmed that higher‐order (especially, prefrontal, inferior parietal and temporal) association cortices have expanded disproportionally in comparison to primary areas in human brain evolution (Avants *et al*., 2006; Van Essen & Dierker, 2007; Rilling, 2014). As observed earlier, due to the underlying network connectivity, the multimodal hub areas of the model spontaneously developed higher CA‐cell density than primary (and secondary) ones. If the ability of the brain to store sets of symbol‐to‐meaning associations relies on the cortex's capacity to develop distinct CA‐circuits that link up specific sensory and motor patterns, an increase in the size of these areas could have represented a crucial evolutionary advantage, as it would have enabled formation and storage of larger numbers of associative circuits while maintaining a low probability of cross‐talk between them. The importance of the relatively larger expansion of higher‐association regions in the human compared with the non‐human primate brain in explaining the emergence of uniquely human linguistic and cognitive capacities has been postulated in the past (Deacon, 1997; Fuster, 1997; Preuss, 2004; Binder *et al*., 2009; Binder & Desai, 2011). Here, a first, putative, cortical‐level mechanistic explanation for this well‐documented evolutionary trend is offered.

### Summary and concluding remarks

Neurocognitive semantic theories propose that word meaning is grounded in the perception and action systems of the human brain (Barsalou, 2008; Pulvermüller & Fadiga, 2010; Glenberg & Gallese, 2012; Pulvermüller, 2013). Using a novel neurocomputational model incorporating basic features of cortical anatomy and function of relevant primary, secondary sensorimotor and higher‐order association areas in the frontal, temporal and occipital lobes, we attempt to elucidate the cortical mechanisms underlying such grounding processes and their consequences at the neurobiological representational level. In particular, the simulations show that Hebbian learning mechanisms at work within specific neuroanatomical structures are sufficient to support the formation of widely distributed lexico‐semantic circuits exhibiting category‐specific cortical topography and associating auditory‐articulatory patterns with semantic information coming from the senses and the motor system. The model is the first computational account able to integrate key experimental observations about: (1) the presence of category‐specific effects in modality‐preferential sensory or motor systems (Pulvermüller & Fadiga, 2010; Meteyard *et al*., 2012); and (2) the emergence and category‐general, ‘across‐the‐board’ character of a range of semantic hubs in multimodal frontal, temporal and parietal cortices, consistently implicated in the processing of all types of meaning (Price, 2000; Patterson *et al*., 2007; Binder & Desai, 2011).

Linking cellular‐level mechanisms with system‐level behaviour, this work offers a novel neurobiological account of conceptual grounding in the brain able to reconcile and explain existing data about different roles of distinct cortical areas during word comprehension processes, providing further computational evidence in support of an action‐perception theory of semantic learning.

## Appendix A Full model specification

Each of the 12 simulated areas (Fig. 1B) was implemented as two layers of artificial neuron‐like elements (‘cells’), 625 excitatory and 625 inhibitory, thus resulting in 15 000 cells in total. Each excitatory cell ‘*e*’ can be considered the network equivalent of a local cluster, or column, of approximately 25 000 real excitatory cortical neurons, that is pyramidal cells, while its twin inhibitory cell ‘*i*’ (Fig. 1C) models the cluster of inhibitory interneurons situated within the same cortical column (Wilson & Cowan, 1972; Eggert & van Hemmen, 2000). The activity state of a cell *e* is uniquely defined by its membrane potential *V*(*e*,* t*), representing the average of all the postsynaptic potentials within neural pool (cluster) *e* at time *t*, and governed by the following equation:

(A1) $$\tau \cdot \frac{dV(e,t)}{dt}=-V\left(e,t\right)+k_{1}\left(V_{\mathrm{In}}\left(e,t\right)+k_{2}\eta \left(e,t\right)\right)$$

where *V*_In_(*e*,* t*) is the net input to cell *e* at time *t* (sum of all inhibitory and excitatory postsynaptic potentials acting upon cluster *e* – I/EPSPs; inhibitory synapses are given a negative sign – plus a constant baseline value *V*_b_), τ is the membrane's time constant, *k*_1_, *k*_2_ are scaling constants and η(*e*,* t*) is a white noise process with uniform distribution over [−0.5, 0.5]. Note that noise is an inherent property of each model cell, intended to mimic the spontaneous activity (baseline firing) of real neurons. Therefore, noise was constantly present in all areas, in equal amounts.^1^

The output (transformation function) of an excitatory cell *e* at time *t* is defined as:

(A2) $$O\left(e,t\right)=\left\{\begin{array}{cc} 0 & \text{if}\;V(e,t)⩽\varphi \\ \left(V(e,t)-\varphi \right) & .55pc\text{if}\;0<(V(e,t)-\varphi )⩽1\\ 1 & .55pc\text{otherwise} \end{array}\right.$$

*O*(*e*,*t*) represents the average (graded) firing rate (number of action potentials per time unit) of cluster *e* at time *t*; it is a piecewise‐linear sigmoid function of the cell's membrane potential *V*(*e*,* t*), clipped into the range [0, 1] and with slope 1 between the lower and upper thresholds φ and φ* *+ 1. The output *O*(*i*,*t*) of any inhibitory cell *i* is 0 if *V*(*i*,* t*) < 0, and *V*(*i*,*t*) otherwise. In excitatory cells, the value of the threshold φ in Eqn A2 varies in time, tracking the recent mean activity of the cell so as to implement neuronal adaptation (Kandel *et al*., 2000). Thus, stronger activity leads to a higher threshold in subsequent time steps. More precisely,

(A3) φ(e,t)=α·ω(e,t)

where ω(*e*,*t*) is the time‐average of cell *e*'s recent output and α is the ‘adaptation strength’. For an excitatory cell *e*, the approximate time‐average ω(*e*,*t*) of its output *O*(*e*,*t*) is estimated by integrating the linear differential equation Eqn A4.1 below with time constant τ_*A*_, assuming initial average ω(*e*, 0) = 0:

(A4.1) $$\tau_{A}\cdot \frac{d\omega (e,t)}{dt}=-\omega \left(e,t\right)+O\left(e,t\right)$$

Local (lateral) inhibitory connections (Fig. 1C) and area‐specific inhibition are also implemented, realising, respectively, local and global competition mechanisms (Duncan, 1996, 2006), and preventing activation from falling into non‐physiological states (Braitenberg & Schüz, 1998). More formally, in Eqn A1 the input *V*_In_(*e*,*t*) to all excitatory cells of the same area includes an area‐specific (‘global’) inhibition term *k*_*S*_· ω_*S*_(*e*,* t*), subtracted from the total sum of the I/EPSPs postsynaptic potentials *V*_In_ in input to the cell, with ω_*S*_(*e*,* t*) defined by:

(A4.2) $$\tau_{S}\cdot \frac{d\omega_{S}\left(e,t\right)}{dt}=-\omega_{S}\left(e,t\right)+\sum_{e\in \mathrm{area}}O\left(e,t\right)$$

The low‐pass dynamics of the cells [Eqns A1, A2, A4.1–2] are integrated using the Euler scheme with step size Δ*t*, where Δ*t* = 0.5 ms.

Excitatory links within and between (possibly non‐adjacent) model areas are established at random and limited to a local (topographic) neighbourhood; weights are initialized at random, in the range [0, 0.1]. The probability of a synapse to be created between any two cells falls off with their distance (Braitenberg & Schüz, 1998) according to a Gaussian function clipped to 0 outside the chosen neighbourhood (a square of size *n *= 19 for excitatory and *n *= 5 for inhibitory cell projections). This produces a sparse, patchy and topographic connectivity, as typically found in the mammalian cortex (Amir *et al*., 1993; Kaas, 1997; Braitenberg & Schüz, 1998; Douglas & Martin, 2004).

The Hebbian learning mechanism implemented simulates well‐documented synaptic plasticity phenomena of long‐term potentiation (LTP) and depression (LTD), as implemented by Artola, Bröcher and Singer (Artola *et al*., 1990; Artola & Singer, 1993). This rule, which covers both ‘true’ Hebbian co‐occurrence (‘what fires together wires together’) as well as ‘anti‐Hebb’ (‘neurons out of sync delink’) plasticity, provides a realistic approximation of known experience‐dependent neuronal plasticity and learning (Rioult‐Pedotti *et al*., 2000; Malenka & Bear, 2004; Finnie & Nader, 2012). In the model, the continuous range of possible synaptic efficacy changes was discretized into two possible levels, +Δ*w* and −Δ*w* (with Δ*w *<< 1 and fixed). Following Artola *et al*., we defined as ‘active’ any link from an excitatory cell *x* such that the output *O*(*x*,* t*) of cell *x* at time *t* is larger than θ_pre_, where θ_pre_ ∈ [0, 1] is an arbitrary threshold representing the minimum level of presynaptic activity required for LTP to occur. Thus, given any two cells *x* and *y* connected by a synaptic link with weight *w*_*t*_(*x*,* y*), the new weight *w*_*t*+1_(*x*,* y*) is calculated as follows:

(A5) $$w_{t+1}\left(x,y\right)=\left\{\begin{array}{cc} w_{t}\left(x,y\right)+\Delta w & \text{if}\;\;O\left(x,t\right)>\theta_{\mathrm{pre}}\;\text{and}\;V\left(y,t\right)>\theta_{\mathrm{post}}\;\left(\text{LTP}\right)\\ w_{t}\left(x,y\right)-\Delta w & \text{if}\;O\left(x,t\right)⩽\theta_{\mathrm{pre}}\;\text{and}\;V\left(y,t\right)>\theta_{\mathrm{post}}\;\left(\text{LTD}\right)\\ w_{t}\left(x,y\right) & \text{otherwise} \end{array}\right.$$

Typical parameter values used during the simulations are as follows:

**Table:**

Eqn A1	Time constant (excitatory cells):	τ = 2.5 (simulation time‐steps)
	Time constant (inhibitory cells):	τ = 5 (simulation time‐steps)
	Scaling factor:	*k*_1_ = 0.01
	Baseline potential	*V*_b_ = 0
	Noise scaling factor	*k*_2_ = 25∙√48
	Global inhibition strength	*k*_*S*_ = 65
	(during training):	*k*_*S*_ = 95
Eqn A3	Adaptation strength:	α = 0.01
Eqns A4.1–2	Average output time constant (for adaptation mechanism):	τ_*A*_ = 10 (simulation time‐steps)
	Global inhibition time constant:	τ_*S*_ = 12 (simulation time‐steps)
Eqn A5	Postsynaptic potential threshold required for synaptic change:	θ_post_ = 0.15
	Presynaptic output activity required for LTP:	θ_pre_ = 0.05
	Learning rate:	Δ*w* = 0.0008
